# Research by Psychiatric Trainees and Early Career Psychiatrists—Results of a Survey From 34 Countries in Europe

**DOI:** 10.3389/fpsyt.2021.718669

**Published:** 2021-09-10

**Authors:** Katja Koelkebeck, Olivier Andlauer, Marton Asztalos, Nikolina Jovanovic, Olga Kazakova, Sean Naughton, Maja Pantovic-Stefanovic, Florian Riese, Mariana Pinto da Costa

**Affiliations:** ^1^Department of Psychiatry and Psychotherapy, Medical Faculty, LVR-Hospital Essen, University of Duisburg-Essen, Essen, Germany; ^2^Center for Translational Neuro- and Behavioral Sciences, University Duisburg-Essen, Essen, Germany; ^3^Early and Quick Intervention in Psychosis (EQUIP), East London NHS Foundation Trust, Donald Winnicot Centre, London, United Kingdom; ^4^Centre for Psychiatry, Wolfson Institute of Preventive Medicine, Barts and the London School of Medicine and Dentistry, Queen Mary University of London, London, United Kingdom; ^5^Aalborg University Hospital, Psychiatry, Aalborg, Denmark; ^6^Unit for Social and Community Psychiatry, World Health Organization (WHO) Collaborating Centre for Mental Health Services Development, Barts and the London School of Medicine and Dentistry, Queen Mary University of London, London, United Kingdom; ^7^Psychiatric Clinic of Minsk City, Minsk, Belarus; ^8^Rotunda Hospital, Dublin, Ireland; ^9^Department for Affective Disorders, Clinic of Psychiatry, University Clinical Center of Serbia, Belgrade, Serbia; ^10^University Research Priority Program “Dynamics of Healthy Aging”, University of Zurich, Zurich, Switzerland; ^11^South London and Maudsley NHS Foundation Trust, London, United Kingdom; ^12^Institute of Psychiatry, Psychology and Neuroscience, King's College London, London, United Kingdom; ^13^Institute of Biomedical Sciences Abel Salazar (ICBAS), University of Porto, Porto, Portugal

**Keywords:** psychiatric trainees, early career psychiatrists, research, barriers, facilitators

## Abstract

Clinical psychiatric practice should be intricately linked with research work. Although psychiatric trainees and early career psychiatrists (ECPs) are in the frontline of clinical services, little is known about how much access they have to research opportunities. A semi-structured questionnaire of 35 questions—exploring research goals achieved, facilitators and barriers as well as personal context—was sent to psychiatric trainees and ECPs across Europe. The survey was disseminated through the local committees of the main professional psychiatric societies in Europe. A total of 258 individuals working in 34 European countries participated. The majority (69.8%) were psychiatric trainees within training in adult psychiatry. Most participants (69.0%) were highly interested in research, but faced major obstacles toward their research activities, such as lack of time and funding. They were highly satisfied with mentoring and publishing papers. Only half of the participants, however, had already published a scientific article, and only a few have been able to contribute to randomized clinical trials (20.9%). A large proportion of participants (87.2%) reported to conduct research after or during a mixture of working hours and after working hours. Only one tenth ever received a grant for their work. These findings highlight that the key barriers for the performance of research are lack of time and funding. Psychiatric trainees and ECPs are motivated to perform research but need support and regular opportunities.

## Introduction

A key part of a clinical academic education is to participate in research activities (1, 2). According to the European Psychiatric Association (EPA) early career psychiatrists (ECPs) are under 40 years old and/or within 5 years of finishing their specialty training (3). Former research has showed that ECPs engage in research activities less often than early career doctors of other medical specialties (4). This might be due to different challenges, including a lack of time for research activities (1, 5–7), insufficient funding (6, 8–10) and less access to scientific literature and internet databases in some European countries (11). In psychiatry, obstacles as lack of appreciation of the field, fewer research funding from pharmaceutical companies and stigmatization of psychiatric research might be some of the obstacles that specifically ECPs face (12). Few resources to conduct research in the home country might lead psychiatric trainees and ECPs to move to higher resourced countries, in a trend so-called “brain-drain” (13, 14).

A recent review on the barriers and facilitators for ECPs to conduct research reported data from small studies covering only eight countries (USA, Canada, Saudi Arabia, UK, Croatia, France, Portugal, Serbia), of which the majority were English-speaking (12). However, little is known about the wider barriers and facilitators for psychiatric trainees and ECPs to conduct research across the European continent. A more representative structured evaluation of the situation for psychiatric trainees and ECPs across Europe is necessary to help formulate strategies for fostering pan-European research activity.

In this article we aimed to identify the perceived research barriers and facilitators among psychiatric trainees and ECPs throughout the European continent.

## Materials and Methods

### Study Design

We conducted a cross-sectional survey targeting psychiatric trainees and ECPs in European countries. The study was designed and conducted as a collaboration by two organizations, namely the Early Career Psychiatrist Committee of the European Psychiatric Association (ECPC-EPA) and the Research Working Group of the European Federation of Psychiatric Trainees (EFPT). These organizations have significant experience of conducting research studies across Europe (13–19).

### Instrument and Its Development

A self-administered, anonymous questionnaire in surveymonkey in English was used. The questionnaire consisted of a total of 35 questions and its content included the key areas identified in the literature (1, 5, 6, 20–24). Twenty-five questions covered: (i) information about research carried out at present, (ii) research goals achieved, (iii) perceived facilitators and barriers to research (i.e., participants were asked to specify time spent in research, payment of research education and funding, whether research training is part of their curriculum and whether it is possible to perform research during working hours). Ten questions concerned socio-demographics such as age, gender, employment status, marital status, education, number of children under the age of 18 and current working conditions (country of origin, whether they presently work in their country of origin, rural vs. urban area, in- vs. outpatient service, university vs. non-university sector). The questionnaire included five-point Likert scale questions, multiple-choice questions or in some items, more than one answer could be chosen and open-ended questions.

The questionnaire was initially piloted with 10 psychiatric trainees and ECPs to check the comprehensibility of the questions and to estimate the time required to complete the questionnaire. The survey was then subject to minor refinements based on this feedback. The mean duration to complete the final survey version was 13 min.

### Data Collection and Handling

The study questionnaire was disseminated through the mailing lists of the EPA-ECPC and the EFPT, via local professional societies of psychiatry and through personal contacts in a snowball sampling. The only inclusion criterion was to be a psychiatric trainee or an ECP in Europe (based on self-declaration).

The survey was approved by the Board of the European Psychiatric Association (EPA). Participants were informed that the questionnaire was anonymous and that personal data were protected. Informed consent was obtained by participants filling the questionnaire.

The authors assert that all procedures contributing to this work comply with the ethical standards of the relevant national and institutional committees on human experimentation and with the Helsinki Declaration of 1975, as revised in 2008.

This study did neither involve a prospective evaluation nor involve animals or vulnerable subjects, e.g., patients. The research did not impose risks, harm or disadvantage on the participants, assessing anonymous data from competent adults only. Ethical approval was, according to the procedures in comparable cases [e.g., (17)] and in accordance with §15/1 of the German professional codex of physicians in its current version therefore not necessary (https://www.bundesaerztekammer.de/fileadmin/user_upload/downloads/pdf-Ordner/MBO/MBO-AE_EN_2018.pdf).

### Data Analysis

SPSS V27 (IBM) was used for statistical analysis. Descriptive statistics were used to report the frequencies and percentages of the categorical variables. Numerical variables are reported as means and standard deviations (SD). The qualitative data from the open-ended questions were analyzed searching for common themes, and the most relevant comments were reported in the respective tables. To provide information about gender-related differences, we computed non-parametric tests for independent samples, comparing the medians.

## Results

### Sample

In total 308 people responded to the survey. From these, 10 people worked in countries outside Europe and 30 indicated no country of employment, and these were hence excluded. Those who did not answer the question regarding their trainee status (*N* = 2) or indicated a status other than psychiatric trainee or ECP (*N* = 2) were excluded. Those participants that indicated being of other specialties than psychiatry (*N* = 6) were also excluded.

The final study sample of 258 participants were on average 31.1 (SD: 4.6) years old and the majority were female (*N* = 170; 65.9%). With relation to their work setting, 90 (34.9%) worked in an academic university, mainly in an urban setting (*N* = 249; 96.5%). The socio-demographic details are reported in Table 1.

**Table 1:** Demographic data of study sample.

**Category**	**Total**	**Female**
Age (years, M ± SD)	31.1 ± 4.6 (missing: *N =* 3)	30.6 ± 4.8
Gender (*N*, f/m)	170/88	–
Marital status (*N*, %)	In a relationship: 106 (41.1) Married: 83 (29.8) Single: 73 (27.5) Other: 3 (1.2) Data missing: 8 (0.4)	78 (44.6) 48 (27.4) 48 (27.4) 1 (0.6) 0
With children below the age of 18 years (*N*, %)	None: 200 (76.7) One: 40 (13.9) Two: 13 (5.2) Three: 5 (2.0) Data missing: 8 (2.7)	139 (79.4) 20 (11.4) 9 (5.1) 2 (1.1) 5 (2.9)
Urban/rural setting (*N*, %)	Urban setting: 256 (96.5) Rural setting: 6 (2.3) Data missing: 4 (1.2)	166 (94.9) 5 (2.9) 4 (2.3)
Employment status (*N*, %)	University: 91 (34.9) Unemployed: 11 (3.9) Other (free answers): 16 (16.2) (research, community center, prison, self-employed, etc.)	56 (32.0) 9 (5.1) 12 (7.1)

**Figure 1:**
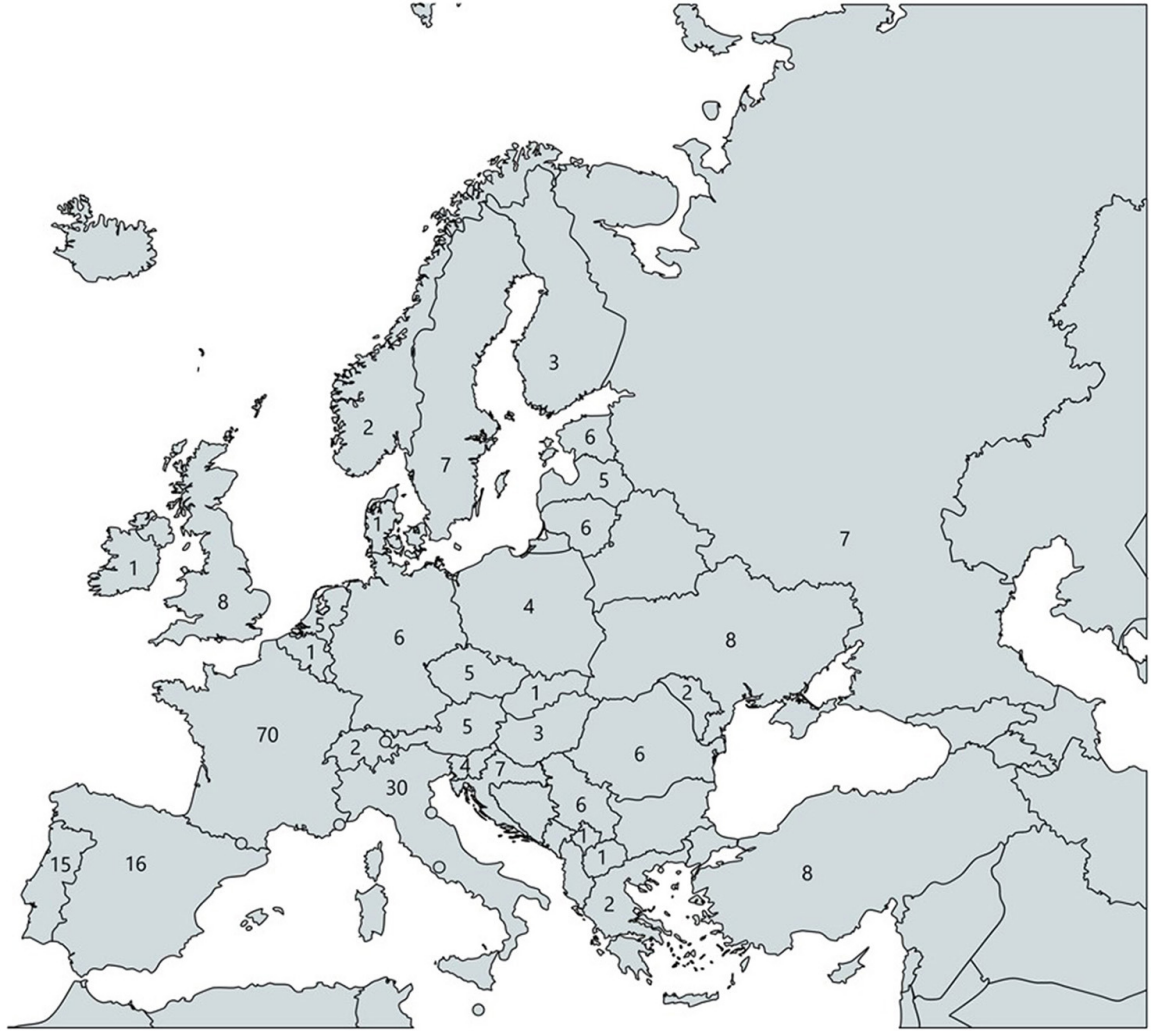
Participants per country of current employment (created with mapchart).

Of the participating respondents, 69.8% were adult psychiatrists (*N* = 180), followed by the specialties of general psychiatry (*N* = 70; 27.1%) and child and adolescent psychiatry (CAP) (*N* = 46; 17.8%). The majority of participants worked in an inpatient service (*N* = 151; 58.5%). Ninety-four (36.4%) participants were ECPs, 156 (60.5%) were psychiatric trainees, 8 indicated other affiliation, but also qualified as psychiatric trainee or ECP according to free texts given (see Table 2). More participants were originated (*N* = 67; 26.0%) and worked (*N* = 70; 27.1%) in France than any other country surveyed (see Figure 1).

**Table 2:** Factors relating to choice of speciality.

**Category**	**Total**	**Female**
Area of work in psychiatry (*N*, %) (more than one answer possible)	Adult psychiatry: 184 (69.8) General psychiatry: 74 (27.1) Child and adolescent psychiatry: 48 (17.8) Addiction psychiatry: 24 (8.9) Liaison psychiatry: 16 (5.8) Old age psychiatry: 15 (5.8) Forensic psychiatry: 6 (2.3) Other (psychosexual, neuropsychiatry, etc.): 1 (0.4)	113 (64.7) 48 (27.4) 35 (20.0) 10 (5.7) 9 (5.1) 13 (7.4) 2 (1.1) 0
Inpatient/outpatient service (*N*, %)	Inpatient service: 168 (58.5) Outpatient service: 98 (36.4)	108 (58.3) 67 (38.3)
Completed duration of employment/training in psychiatry (years, M ± SD)	ECP: 2.8 ± 2.2 Trainee: 2.7 ± 1.4 Data missing: *N =* 13	2.6 ± 1.7 2.5 ± 1.5 0
Total duration of postgraduate training program (years, M ± SD)	ECP: 4.3 ± 2.1 Trainee: 2.4 ± 1.6 Data missing: *N =* 22	4.2 ± 2.2 2.6 ± 1.6 0
Contact with psychiatry before graduation from medical school (*N*, %)	Yes: 205 (78.3)	132 (75.4)
If yes, by way of (*N*, %) (more than one answer possible)	Internship: 110 (41.9) Lectures: 94 (36.0) Research work: 61 (23.3) Clinical placement: 61 (22.9) Voluntary work: 49 (18.6) Professional education (e.g., nurse): 10 (3.9) Other (free answers): 7 (2.7) Balint group, relatives, own treatment	74 (42.3) 63 (36.0) 34 (19.4) 38 (21.7) 36 (20.6) 8 (4.6) 3 (1.8)
Duration of contact (*N*, %)	More than 6 months: 73 (27.9) 1–3 months: 58 (22.1) 3–6 months: 37 (14.3) 1–2 weeks: 33 (12.4) <1 week: 6 (1.9) Data missing: 2 (1.3)	43 (24.6) 41 (23.4) 28 (16.0) 17 (9.7) 4 (2.3) 0
Reason for choosing psychiatry (*N*, %) (more than one answer possible)	Interest in psychiatry: 230 (86.4) Interest in psychiatric disorders: 180 (67.4) Interest in human sciences: 156 (59.7) Interst in working with the patient group: 88 (34.1) Research options: 85 (31.8) Familiy history of psychiatric disorders: 37 (14.3) Availabililty of training positions: 24 (8.9) Personal history of psychiatric disorders: 21 (8.1) Financial incentives: 7 (2.7) Other (free answers): 4 (1.6) lifestyle, meeting patients and their families, teamwork, good professors, empathic abilities	150 (85.7) 119 (68.0) 111 (63.4) 65 (37.1) 52 (29.7) 26 (14.9) 12 (6.9) 18 (10.3) 5 (2.9) 4 (2.4)

Some countries, for instance the Scandinavian region (e.g., Finland, Iceland), were less well-represented. France was also the country in which most of the participants were employed. Some participants (*N* = 26, 10.1%) indicated that they did not work in their country of origin. A majority of the participants (*N* = 202; 78.3%) had experience with the specialty before graduation through internships (*N* = 108; 41.9%), lectures (*N* = 93; 36.0%) and clinical placements (*N* = 59; 22.9%) but also through research work (*N* = 60; 23.3%). The majority of participants had more than 1 month exposure to psychiatry before graduation, and at least a quarter more than 6 months (*N* = 72; 27.9%). The reasons to choose psychiatry as a career were interest in psychiatry (*N* = 223; 86.4%), in mental disorders (*N* = 174; 67.4%) or human sciences (154; 59.7%) (see Table 2).

### Research Interest and Motivation of Trainees and ECPs

The majority of participants (*N* = 217, 84.1%) reported to have at one point conducted research (for details, see Table 3).

**Table 3:** Results of research activities.

**Category (N, %)**	**Total**	**Female**
Ever performed research	Yes: 222 (84.1)	145 (82.9)
Postgraduate research training	None: 163 (61.2) Master: 58 (22.1) PhD: 34 (13.2) Other (Dr. med., MD, etc.): 9 (3.1) Data missing: 2 (0.4)	113 (64.6) 32 (18.3) 23 (13.1) 5 (2.9) 2 (1.1)
Working toward a postgraduate degree	None: 174 (66.3) PhD: 64 (24.0) Master: 13 (5.0) Other (MD, specific research program…): 14 (4.7) Data missing: 1 (0.4)	123 (70.3) 34 (19.4) 10 (5.7) 7 (4.0) 1 (0.6)
Level of interest in research (beginning of career in psychiatry)	Very low/low: 46 (17.0) Neutral: 59 (22.5) Strong/very strong: 156 (58.9) Data missing: 5 (1.6)	33 (18.8) 37 (21.1) 100 (57.2) 5 (2.9)
Level of interest in research (currently)	Very low/low: 33 (12.8) Neutral: 48 (18.2) Strong/very strong/:184 (69.0) Data missing: 1 (0.4)	23 (13.1) 35 (20.0) 116 (66.3) 1 (0.6)

Only a minority of respondents had already completed (*N* = 34; 13.2%) or worked toward (*N* = 62; 24.0%) a postgraduate research degree (PhD). A larger proportion of participants have indicated that they had a strong or very strong (*N* = 152; 58.9%) motivation to conduct research before they started their career in psychiatry as well as at present (*N* = 186; 69.0%).

Most participants agreed or strongly agreed (*N* = 225; 86.2%) that research is not only essential for training and practice of medicine, but also for career advancement (*N* = 197; 76.3%). Moreover, a significant portion of participants (*N* = 228; 88.4%) considered that training in research methods should be incorporated into the training curricula (see Table 4).

**Table 4:** Attitudes toward research. (*N*, %), second value indicates females.

**Category**	**Strongly disagree/** **disagree**	**Neutral**	**Strongly agree/** **agree**	**Missing**
Research is essential in the practice of medicine	9 (3.5)/5 (2.9)	24 (9.3)/13 (7.4)	232 (87.2)/156 (89.2)	1 (0.4)/1 (0.6)
Trainees should participate in research	9 (3.5)/6 (3.4)	30 (11.2)/18 (10.3)	225 (84.9)/149 (85.1)	2 (0.8)/2 (1.1)
Training research methodology should be part of the training curriculum	6 (2.3)/4 (2.3)	23 (8.9)/17 (9.7)	235 (88.4)/152 (83.8)	2 (0.8)/2 (1.1)
Research promotes critical thinking	4 (1.2)/2 (1.2)	12 (4.7)/6 (3.4)	247 (93.4)/165 (94.3)	3 (1.1)/2 (1.1)
Research improves healthcare	2 (0.8)/2 (1.1)	23 (8.5)/13 (7.4)	236 (89.1)/157 (89.7)	5 (1.9)/3 (1.7)
Research helps further my career	13 (5.1)/10 (5.7)	46 (17.4)/30 (17.1)	203 (76.3)/132 (75.4)	4 (1.5)/3 (1.7)

### Conditions of Research Work and Research Outcome in Trainees and ECPs

#### Research Activities

Within the topics of research indicated, social psychiatry (*N* = 54; 20.9%), epidemiology (*N* = 43; 16.7%) and pharmacology (*N* = 45; 17.4%) were the most frequent topics, genetics (*N* = 3; 1.2%) and cell studies (*N* = 5; 1.9%) were the least. Participants had contributed to research through a literature review (*N* = 185; 71.7%), data entry (*N* = 159; 61.6%), data analysis (*N* = 128; 49.6%), concept and design of studies (*N* = 135, 52.3%), communication with editorial offices (*N* = 102; 39.5%) and redrafting articles after review (*N* = 87; 33.7%). For more details, see Table 5.

**Table 5:** Research activities.

**Category**	**Total**	**Female**
Research area (*N*, %)	Social psychiatry: 56 (20.9) Pharmacological: 46 (17.4) Epidemiological: 44 (16.7) Psychotherapy: 22 (8.5) Molecular/biochemical: 22 (8.5) Health system/service evaluation: 20 (7.8) Imaging: 12 (4.7) Cell study: 5 (1.9) Genetics: 3 (1.2) Social interventions: 2 (0.8) Other (psychosexual, stigma, psycho-oncology….): 13 (4.7) None: 2 (0.8) Data missing: 19 (6.2)	36 (20.6) 26 (14.9) 31 (17.7) 16 (9.1) 16 (9.1) 16 (9.1) 4 (2.3) 3 (1.7) 1 (0.6) 2 (1.1) 9 (5.1) 2 (1.1) 13 (7.4)
When is research mainly performed (*N*, %)	A mixture of during and after working hours: 123 (46.5) After working hours: 108 (40.7) In dedicated time slots for research only: 12 (4.7) During working hours: 10 (3.5) Other: 7 (2.7) Data missing: 6 (1.9)	76 (43.4) 74 (42.3) 9 (5.1) 5 (2.9) 6 (3.4) 5 (2.9)
Contributions to research (*N*, %) (more than one answer possible)	Literature review: 187 (71.7) Data entry: 164 (61.6) Concept/design of research: 137 (52.7) Drafting of manuscripts: 134 (51.9) Data analysis: 131 (49.6) Generate new research questions: 125 (47.3) Perform experiments: 110 (42.2) Writing a proposal: 108 (41.1) Preparation for publishing (cover letters, etc.): 103 (39.5) Redrafting (incorporating review): 87 (33.7) Other (free answers): 8 (3.1) interviews, distribution of information, etc.	118 (67.4) 99 (56.6) 76 (43.4) 84 (48.6) 79 (45.1) 71 (40.6) 66 (37.7) 65 (37.1) 60 (34.3) 48 (27.4) 7 (4.1)
Types of research projects (*N*, %) (more than one answer possible)	Literature reviews: 142 (54.7) Case reports: 130 (49.6) Questionnaire /survey-based: 116 (44.6) Retrospective clinical studies: 80 (30.6) Cross-sectional studies: 76 (27.9) Prospective clinical studies: 70 (27.1) Randomized clinical trials: 54 (20.9) Qualitative/semi-structured interviews: 54 (20.9) Systematic review: 46 (17.8) Meta-analysis: 31 (12.0) Monitoring, surveillance: 27 (10.5) Registry studies: 15 (5.8) Other (free answers): 7 (2.7) biological, pre-clinical, interventions	92 (52.6) 93 (53.1) 77 (44.0) 48 (27.4) 40 (22.9) 35 (20.0) 30 (22.9) 34 (19.4) 29 (19.4) 18 (16.6) 14 (10.3) 8 (4.6) 7 (4.1)
Experience of presenting results (*N*, %) (more than one answer possible)	Oral presentation: 149 (57.0) Poster Presentation: 144 (55.0) Research paper publication: 99 (38.4) International journal: 81 (30.2) Peer-reviewed: 74 (28.3) Print publication (e.g. thesis): 65 (25.2) Never published: 57 (21.7) National journal: 60 (22.9) Book chapter: 34 (13.2) Non peer-reviewed: 18 (7.0) Newspaper: 14 (5.4) Other (free answers): 13 (5.0) e.g. in preparation	89 (50.9) 84 (48.0) 55 (31.4) 52 (29.7) 35 (20.0) 38 (21.7) 41 (23.4) 40 (22.9) 20 (11.4) 10 (5.2) 9 (5.1) 13 (7.7)
Best paper (credits) (*N*, %)	First author: 111 (42.2) Co-author: 76 (28.7) Last author: 5 (1.9) Other (not published yet, not credited, ….): 22 (8.5) Data missing: 52 (18.6)	70 (40.0) 48 (27.4) 3 (1.7) 19 (10.9) 35 (20.0)
The highest impact factor of a published journal in the field (mean ± SD)	6.9 (95% CI: 5.1–8.8) Range: 0.4–34.0 (**N* =* 61)	6.7 (95% CI: 4.3–9.5) 0.4–33.6 (*N =* 32)
Grant received (*N*, %)	Yes: 60 (22.6) Data missing: 14 (5.3)	32 (18.3) 11 (6.3)
What kind of grant received (*N*, %) (more than one answer possible)	Institutional grant: 31 (12.0) National grant: 25 (9.7) Grant by foundation: 15 (5.8) International grant: 5 (1.9) Industry-sponsored: 4 (1.2) Other (free answers): 5 (1.9) EFPT research prize, other specialty funding	17 (9.7) 14 (8.0) 9 (5.1) 3 (1.7) 1 (0.6) 5 (2.9)
Financial support for presentation (*N*, %)	Yes: 47 (17.4) Data missing: 18 (6.2)	26 (14.9) 13 (7.4)
Travel allowance (*N*, %) (more than one answer possible)	Institution: 31 (11.6) National funding: 13 (5.0) Industry funding: 12 (4.7) Other (free answers): 8 (3.1) ECNP, fellowship, international funding, congress committee fellowship	15 (8.6) 6 (3.4) 5 (2.9) 8 (4.7)
Feel sufficiently experienced to perform own project (*N*, %)	Yes: 85 (31.0) Data missing: 12 (4.5) Other (free answers): 92 (35.7) depends, no interest, with enough support……	42 (24.0) 11 (6.3) 68 (40.0)
Proficiency in the English language (*N*, %)	Full proficiency: 122 (45.3) Intermediate proficiency: 112 (43.0) Novice proficiency: 22 (8.1) Native speaker/bilingual: 10 (3.5) Data missing: 22 (8.1)	71 (40.6) 85 (48.6) 13 (7.4) 6 (3.4) 0

#### Publications, Presentations, and Grants

Participants reported experience in various different study designs, such as literature review (*N* = 141; 54.7%), case report (*N* = 128; 49.6%), survey (*N* = 115; 44.6%), and randomized clinical trial (*N* = 54; 20.6%). Most had experience presenting in oral (*N* = 147; 57.0%) or poster (*N* = 142; 55.0%) format, while only 38.4% (*N* = 99) of participants had published a paper. In their best paper, several participants were credited as first authors (*N* = 109; 42.2%).

Among those who have indicated an impact factor of their research, the median was 4.6, with the lowest impact factor 0.4 (“unknown journal”) and the highest 34.0 (“Science”). About a quarter of participants (*N* = 60, 23.3%) had received a grant for the study that they are or were working on. Of those participants, who indicated that they had received a grant, institutional grants were indicated most frequently (*N* = 31; 12.0%), followed by national grants (*N* = 25; 9.7%). The same was true for presentation of results: institutional grants (*N* = 30; 11.6%) being the single biggest source of funding for the presentation of results, such as conferences. For more details, see Table 5.

#### Research Working Conditions

**Figure 2:**
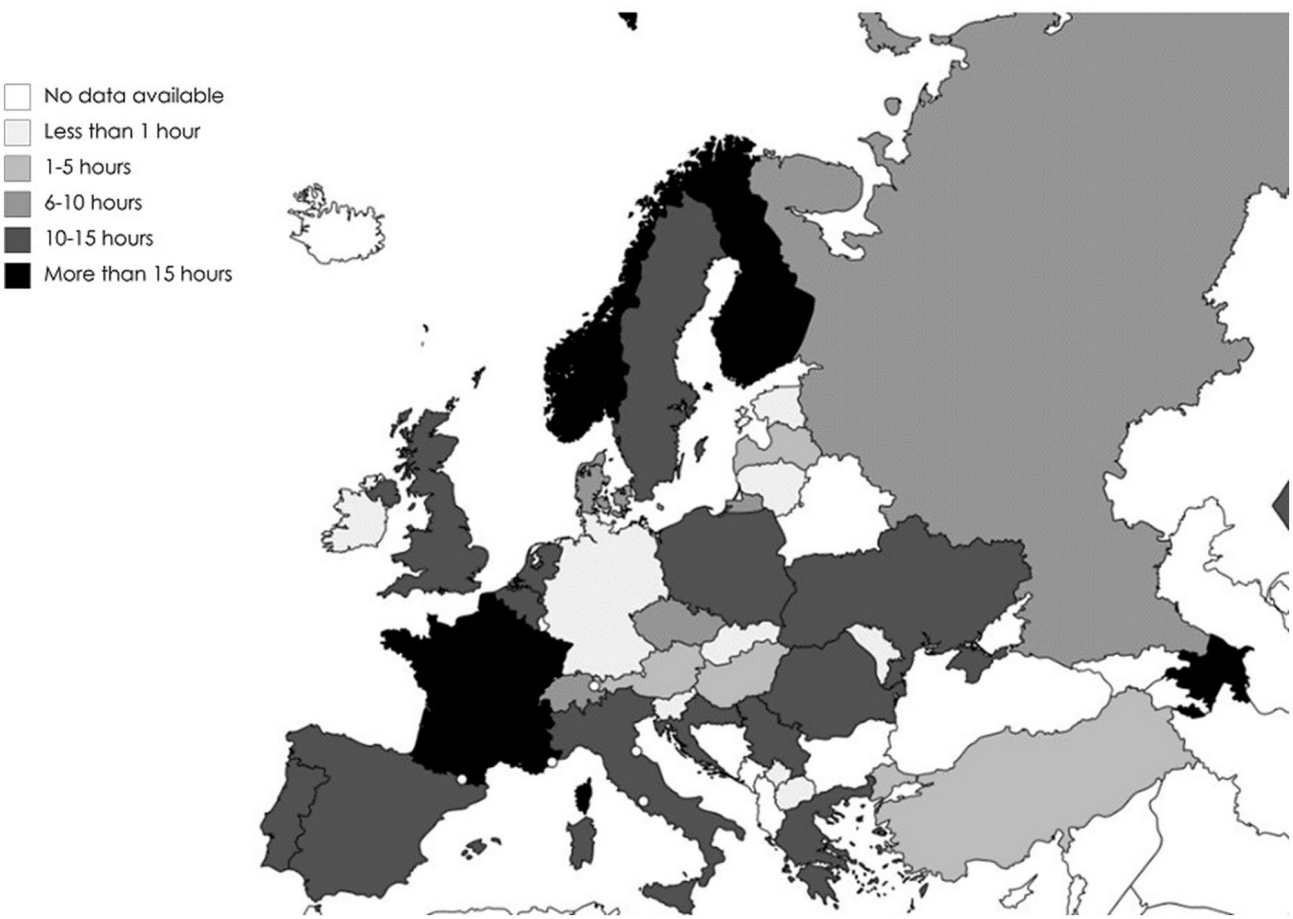
Hours (per week) spent in research per country of employment.

Participants indicated that they conducted research mainly after working hours (*N* = 105; 40.7%) or in a mixture of working hours and after working hours (*N* = 120; 46.5%). A larger proportion was able to work in research 1–5 h per week on average (*N* = 109; 42.2%), whereas 21.7% (*N* = 56) managed to work 6–10 h per week, see also Table 5 and Figure 2.

### Perceived Barriers and Facilitators

**Figure 3:**
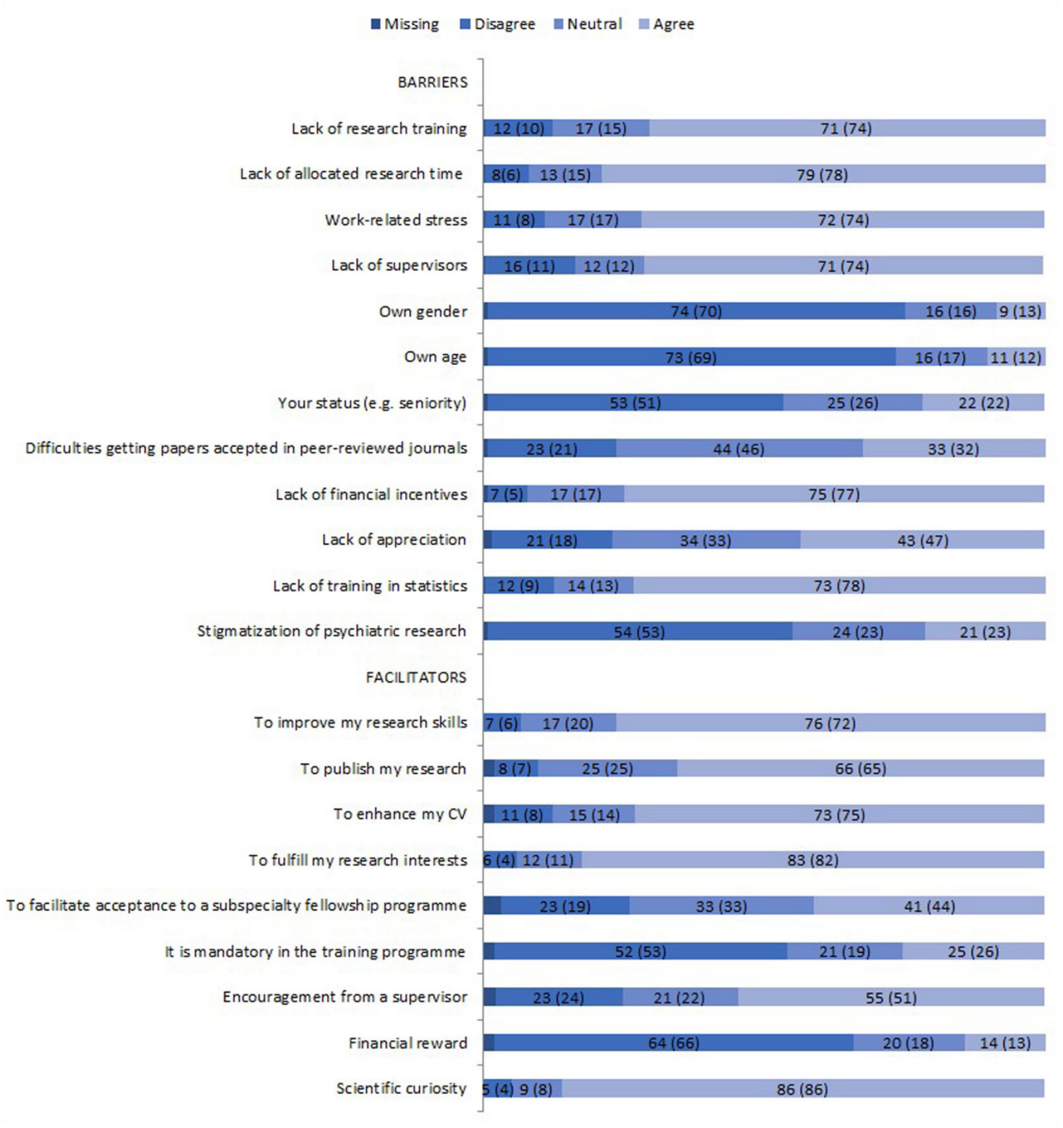
Perceived barriers and facilitators regarding research (percentages). Values for female participants only are given in brackets.

Among the common barriers to performing research, participants mentioned lack of training, lack of funding, lack of allocated time, lack of supervisors and work-related stress. Barriers such as lack of appreciation, gender, age, lower working status, stigmatization of research in psychiatry as well as receiving rejection following paper submission was less frequently indicated (see Figure 3 for details).

With regards to facilitators: scientific curiosity, fulfilling research interest, enhancing the CV, publishing the research and encouragement from a mentor were mentioned. Less frequently mentioned were financial reward, inclusion of research in the training program or research training within the curriculum (see Figure 3 for details).

Only about one third of participants (*N* = 80, 31.0%) felt sufficiently prepared by their previous experience to be able to perform their own research projects. Most of them indicated they did not have enough training or guidance nor time to conduct projects on their own. However, most participants (*N* = 224, 89.2%) indicated that they had sufficient English language proficiency. For details also see Table 5.

### Distribution of Research Resources Across Europe and Research Opportunities per Country of Work

**Figure 4:**
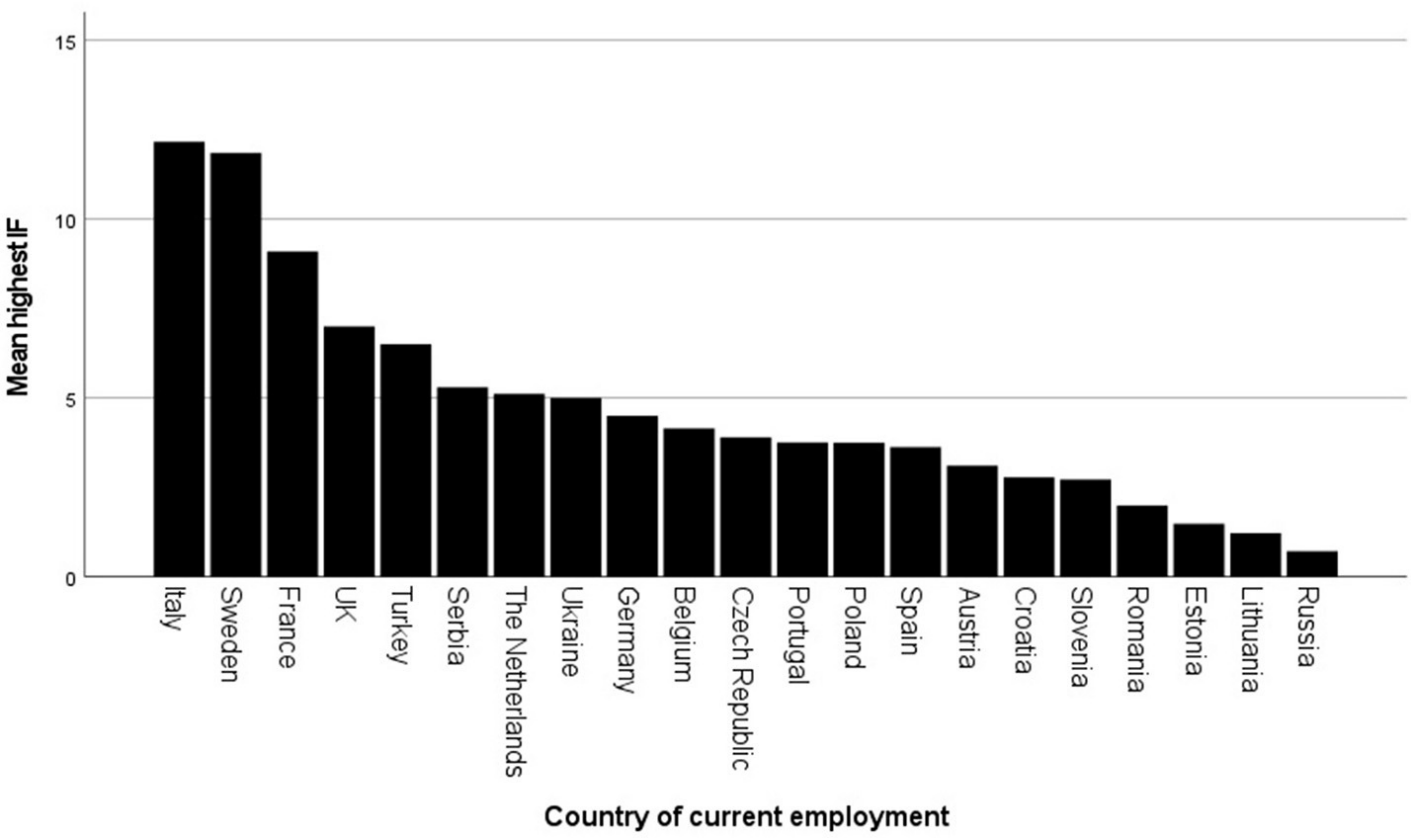
Impact factor (IF, means) of best publication per country of employment.

When looking at the distribution of research resources by country, it is to be stated that regarding time spent in research or highest impact, the Scandinavian and South-Western European countries seem to offer a better basis for research than Eastern European countries (see Figure 4).

Norway, Finland and France were also the countries where participants had the most time for research per week. Slovenian, Slovakian, Macedonian, Ukrainian, Kosovon^1^, but also German and Irish participants indicated the least time to spend on research per week.

### Gender Differences

With relation to gender differences on barriers and facilitators to research, only the barrier “gender” was answered differently by female and male participants (*p* = 0.023). Female participants indicated in 13.2% of cases that they agreed that gender might pose a barrier to research, 16.0% reported a neutral attitude and 69.7% answered they did not agree. Male participants indicated in 82.4% of the cases that they did not agree that there was a gender-associated barrier, 16.7% reported a neutral attitude and none of male participants agreed that gender might pose a barrier. All other aspects remained non-significant. However, female participants felt less competent to conduct research (*p* < 0.001).

## Discussion

### Comparison With the Literature

The results of this cross-sectional European survey are similar to those identified in a previous review of barriers and facilitators of research (12), as well as a review of research activities (11). In particular these findings confirm that higher income countries, such as Sweden or the Netherlands make for stronger research backgrounds, which draws migrants from across Europe (13). However, a previous review primarily compared studies in a single or few countries, which were disproportionately English-speaking. Thus, our present approach draws a more comprehensive picture in Europe, including also countries form Eastern Europe. Nevertheless, reporting of factors which facilitate or impede research activities were fairly similar to previous studies.

In this survey we identified several barriers and facilitators to conduct research. The most prominent ones were work-related stress, lack of allocated research time, training and mentors. Former research had already identified work-related stress as specifically an issue in trainees (15, 16), where the ability to balance work and free time has yet to develop properly. Lack of motivated mentors (6, 25–27), too little allocated research time and lack of research training (26) have also been identified in previous studies, albeit in other contexts.

As was shown in this survey, across participants, highest impact factors were achieved in Sweden, France and Italy. Most of the participants of the Eastern European countries seem to publish in lower-impact journals. This may indicate lower development of research infrastructure in these countries, a preference to publish in the local language or lower acceptance rates for manuscripts from lower-income countries (28). However, only few of the participants were currently working in these countries, and may therefore not be representative.

### Strengths and Limitations

A strength of our survey is that psychiatric trainees or ECPs from 34 different countries participated, sharing their opinions and experiences on research, directly relating to themselves and indirectly to their country. Moreover, we assessed different aspects of research (e.g., funding opportunities, time for research, publication experience) that might help us understand what facilitates or restricts research in certain countries or geographic areas.

One limitation of this study is the small sample and convenience sample with possible bias toward participants interested in research. The number of psychiatric trainees in Europe has been estimated as close to 20,000 (13, 29). This study should therefore not be considered representative of the average psychiatric trainee or ECP in Europe, but may rather reflect the ones motivated to conduct research. Moreover, the large proportion of trainees or ECPs from certain European countries, e.g., France, makes it harder to draw meaningful results for other countries in Europe. Also, due to the unequal geographic distribution of participants, country-specific analyses cannot be performed and were not the focus of this work. In addition, to explore gender barriers was not the main target of this study, and therefore the median comparisons reported might not be an optimal instrument to identify gender differences. Future research should thus explicitly target Eastern-European countries, since they are generally more exposed to brain-drain. Likewise, major migration destination countries for academic reasons such as Switzerland and the UK (13) are under-represented in our study. Equally, future research should further explore potential gender differences in accessing professional development and academic opportunities.

### Future Implications for Practice, Policies and Research

The data gathered through this survey may help to enhance the attention that research—a critical component of high-quality training in psychiatry—receives. This may help to develop European guidelines to foster research in psychiatric trainees and ECPs. Guidelines can be proposed for European-wide training opportunities in research for trainees and ECPs on the basis of the results presented here. Some organizations have already started to spark research activities in early career stages, promoting prizes and fellowships as well as workshops on research, for instance the EPA (EPA Research Prize), the EFPT (EFPT Porto Research Award), the World Psychiatric Association (WPA) (24) as well as the Collegium Internationale Neuropharmacologicum (CINP). Some European countries have also already included research topics in their regular curricula. However, in many countries research is neither a topic in the education of trainees or ECPs nor is protected research time a reality in European countries (12). In particular, to reduce brain-drain in both clinical and research work, resources to conduct research need to improve. This might make countries liable to “brain-drain”—notably those in East Europe—more attractive to talented researchers, whether domestic or foreign (13).

As elsewhere summarized (12), interest in research needs to be sparked early in a career. It is necessary to actively involve psychiatric trainees and ECPs in research projects and encourage them to publish their work (25). Structured information included in training can help reduce worries regarding research (30, 31). Research academies in medical faculties or inclusion of research time in training programs might reduce stress (32). Moreover, specific training curricula (33), research events (34) and supervised activities (35) as well as structured research training (36) might spark interest in future researchers in psychiatry.

As a proportion of female trainees and ECPs seem to perceive a gender gap regarding research, and feel less competent to conduct research, they should be encouraged more explicitly to engage in research activities. Faculties should also be aware of shortcomings in their country, so they can react and prevent “brain-drain.” Lastly, at the national level, research opportunities should be included in the curricula of trainees and ECPs.

When the participants in this study were asked for the most important research topics as recommended by the ROAMER project (37), the top three were: (i) prevention, health promotion and interventions in mental disorders in young people; (ii) causes and development of mental ill-health across the lifespan (including older people); (iii) reducing stigma, and empowering service users and their careers in decisions about their mental health care. The topics that are of relevance to the early career professionals in psychiatry should be encouraged.

Psychiatric trainees and ECPs outside of Europe are called upon to explore if the reality in other countries (in particular in low-to-middle-income countries) is different. More qualitative research with trainees and ECPs with experience of research is needed to further understand their experiences as well as barriers and facilitators at the individual level.

### Conclusions

This study reports the research facilitators and obstacles for psychiatrists in the early stages of their career in Europe. While this first evaluation showed that more allocated time and funding is needed for research, further exploration is still needed at the national, European and global level.

## Data Availability Statement

The raw data supporting the conclusions of this article will be made available by the authors, without undue reservation.

## Ethics Statement

Ethical review and approval was not required for the study on human participants in accordance with the local legislation and institutional requirements. The patients/participants provided their written informed consent to participate in this study.

## Author Contributions

KK, MP-S, NJ, OK, SN, MP, FR, and OA developed the study. KK, OK, and SN assessed data. KK, MA, and MP performed data analyses. KK and MP-S prepared tables and figures. KK and MP wrote the first draft. All authors reviewed the manuscript.

## Conflict of Interest

FR reports personal fees from Merz Pharma and Vifor Pharma, not related to the submitted work. The remaining authors declare that the research was conducted in the absence of any commercial or financial relationships that could be construed as a potential conflict of interest.

## Publisher's Note

All claims expressed in this article are solely those of the authors and do not necessarily represent those of their affiliated organizations, or those of the publisher, the editors and the reviewers. Any product that may be evaluated in this article, or claim that may be made by its manufacturer, is not guaranteed or endorsed by the publisher.

## Footnotes

^1^All references to Kosovo, whether the territory, institutions or population in this text shall be understood in full compliance with United Nations Security Council Resolution 1244 and without prejudice to the status of Kosovo.

## References

[1] Burford C. Attitudes to research among Royal Free psychiatric trainees and consultants. Psychiatr Bull. (1987) 11:254–7. 10.1192/pb.11.8.254

[2] Pinto da Costa M. Early career psychiatrists - history, 2020 and beyond. World Psychiatry. (2020) 19:127–8. 10.1002/wps.20712 31922680 PMC6953585

[3] Pinto da Costa M Kilic O Ismayilova J Mogren T Smirnova D Gondek T. What opportunities do early career psychiatrists have in Europe and beyond? BJPsych Int. (2020) 17:95–6. 10.1192/bji.2020.29 33196700 PMC7609988

[4] Burke JD Pincus HA Pardes H. The clinician-researcher in psychiatry. Am J Psychiatry. (1986) 143:968–75. 10.1176/ajp.143.8.968 3728743

[5] Lewis S. Training matters. The right stuff? A prospective controlled trial of trainees' research. Psychiatr Bull. (1991) 15:478–80. 10.1192/pb.15.8.478

[6] Williams CJ Curran S. Research by senior registrars in psychiatry: lessons to be learned for the specialist registrar grade. Psychiatr Bull. (1998) 22:102–4. 10.1192/pb.22.2.102

[7] Van Effenterre A. Education and training of young psychiatrists: is there time for research? Encephale. (2011) 37:159–61. 10.1016/j.encep.2010.08.008 21703430

[8] Sedvall GC. Internationalization of psychiatric research - the prospective for the European Association of Psychiatrists. Acta Psychiatr Scand. (2002) 105:321–3. 10.1034/j.1600-0447.2002.2e004.x 11942938

[9] Emsley R. Focus on psychiatry in South Africa. Br J Psychiatry. (2001) 178:382–6. 10.1192/bjp.178.4.382 11282826

[10] Patel V Sumathipala A. International representation in psychiatric literature: survey of six leading journals. Br J Psychiatry. (2001) 178:406–9. 10.1192/bjp.178.5.406 11331553

[11] Gama Marques J Pantovic Stefanovic M Mitkovic-Voncina M Riese F Guloksuz S Holmes K. Equal access for all? Access to medical information for European psychiatric trainees. Psychiatry Res. (2016) 238:150–2. 10.1016/j.psychres.2016.02.015 27086225

[12] Koelkebeck K Pantovic Stefanovic M Palumbo C Frydecka D Andlauer O Riese F. Barriers and facilitators to conducting research by early career psychiatrists: a literature review. Global Psychiatry. (2020) 2:135–53. 10.2478/gp-2019-0018

[13] Pinto da Costa M Giurgiuca A Holmes K Biskup E Mogren T Tomori S. To which countries do European psychiatric trainees want to move to and why? Eur Psychiatry. (2017) 45:174–81. 10.1016/j.eurpsy.2017.06.010 28957784

[14] Pinto da Costa M. Workforce migration and brain drain in psychiatry trainees. Eur Psychiatry. (2015) 30(Suppl. 1):89. 10.1016/S0924-9338(15)31831-924908150

[15] Jovanovic N Beezhold J Andlauer O Kuzman MR Podlesek A Hanon C. Burnout among psychiatry residents - The International Psychiatry Resident/Trainee Burnout Syndrome Study (BoSS). Die Psychiatrie. (2009) 6:75–9. 10.5167/uzh-19403

[16] Jovanovic N Podlesek A Volpe U Barrett E Ferrari S Rojnic Kuzman M. Burnout syndrome among psychiatric trainees in 22 countries: risk increased by long working hours, lack of supervision, and psychiatry not being first career choice. Eur Psychiatry. (2016) 32:34–41. 10.1016/j.eurpsy.2015.10.007 26802982

[17] Jauhar S Guloksuz S Andlauer O Lydall G Marques JG Mendonca L. Choice of antipsychotic treatment by European psychiatry trainees: are decisions based on evidence? BMC Psychiatry. (2012) 12:27. 10.1186/1471-244X-12-27 22463055 PMC3337226

[18] Koelkebeck K Andlauer O Jovanovic N Giacco D. Interventions for posttraumatic stress disorder in psychiatric practice across Europe: a trainees' perspective. Eur J Psychotraumatol. (2015) 6:27818. 10.3402/ejpt.v6.27818 26350154 PMC4563100

[19] Rojnic Kuzman M Andlauer O Burmeister K Dvoracek B Lencer R Koelkebeck K. Effective assessment of psychotropic medication side effects using PsyLOG mobile application. Schizophr Res. (2018) 92:211–2. 10.1016/j.schres.2017.04.038 28457773

[20] Strauss GD Yager J Offer D. Research training in psychiatry: a survey of current practices. Am J Psychiatry. (1980) 137:727–9. 7377397 10.1176/ajp.137.6.727

[21] Balon R Singh S. Status of research training in psychiatry. Acad Psychiatr. (2001) 25:34–41. 10.1176/appi.ap.25.1.34

[22] Fitz-Gerald MJ Kablinger A Manno B Carter OS Caldito G Smith S. Psychiatry residents' participation in research: a survey of attitudes and experience. Acad Psychiatr. (2001) 25:42–7. 10.1176/appi.ap.25.1.42

[23] Hauge EM Grønbaek H. [Key issues for the number of publications by Ph.D. graduates in medicine and pharmaceutical sciences]. Ugeskr Laeger. (2009) 171:699–703. 19257995

[24] Parker G. How do research psychiatrists rate? Views Neurosci. (1995) 29:500–3. 10.3109/00048679509064960 8573055

[25] Laliberte V Rapoport MJ Andrew M Davidson M Rej S. Career interests of Canadian psychiatry residents: what makes residents choose a research career? Can J Psychiatry. (2016) 61:86–92. 10.1177/0706743715625952 27253699 PMC4784243

[26] Mitwalli HA Al Ghamdi KM Moussa NA. Perceptions, attitudes, and practices towards research among resident physicians in training in Saudi Arabia. East Mediterr Health J. (2014) 20:99–104. 10.26719/2014.20.2.99 24945558

[27] Pinto da Costa M Guerra C Malta R Moura M Carvalho S Mendonca D. Psychiatry training towards a global future: Trainees' perspective in Portugal. Acta Med Port. (2013) 26:357–60. Available online at: https://www.actamedicaportuguesa.com/revista/index.php/amp/article/view/319/3730 24016644

[28] Yousefi-Nooraie R Shakiba B Mortaz-Hejri S. Country development and manuscript selection bias: a review of published studies. BMC Med Res Methodol. (2006) 6:37. 10.1186/1471-2288-6-37 16879753 PMC1550721

[29] Kuzman MR Giacco D Simmons M Wuyts P Bausch-Becker N Favre G. Psychiatry training in Europe: views from the trenches. Med Teach. (2012) 34:e708–17. 10.3109/0142159X.2012.687481 22646296

[30] Balon R Heninger G Belitsky R. Medical school research pipeline: medical student research experience in psychiatry. Acad Psychiatry. (2006) 30:16–22. 10.1176/appi.ap.30.1.16 16473989

[31] Myint PK MacLullich AM Witham MD. The role of research training during higher medical education in the promotion of academic medicine in the UK. Postgrad Med J. (2006) 82:767–70. 10.1136/pgmj.2006.046227 17101612 PMC2660513

[32] Bartels SJ Lebowitz BD Reynolds CF 3rd Bruce ML Halpain M Faison WE. Programs for developing the pipeline of early-career geriatric mental health researchers: outcomes and implications for other fields. Acad Med. (2010) 85:26–35. 10.1097/ACM.0b013e3181c482cb 20042817 PMC2931586

[33] Posporelis S Sawa A Smith GS Stitzer GL Lyketsos CG. Promoting careers in academic research to psychiatry residents. Acad Psychiatry. (2014) 38:185–90. 10.1007/s40596-014-0037-7 24497181 PMC4128627

[34] Mills LS Steiner AZ Rodman AM Donnell CL Steiner MJ. Trainee participation in an annual research day is associated with future publications. Teach Learn Med. (2011) 23:62–7. 10.1080/10401334.2011.536895 21240786

[35] Cheung G Hatters Friedman S Ng L Cullum S. Supervising trainees in research: what does it take to be a scholarly project supervisor? Australas Psychiatr. (2018) 26:214–9. 10.1177/1039856217726696 28879779

[36] Hamoda HM Bauer MS DeMaso DR Sanders KM Mezzacappa E. A competency-based model for research training during psychiatry residency. Harv Rev Psychiatry. (2011) 19:78–85. 10.3109/10673229.2011.565249 21425936

[37] Fiorillo A Luciano M Del Vecchio V Sampogna G Obradors-Tarrago C Maj M. Priorities for mental health research in Europe: A survey among national stakeholders' associations within the ROAMER project. World Psychiatry. (2013) 12:165–70. 10.1002/wps.20052 23737426 PMC3683269

